# Preparation, Physical Properties, and Applications of Water-Based Functional Polymer Inks

**DOI:** 10.3390/polym13091419

**Published:** 2021-04-27

**Authors:** Edgar Gutiérrez-Fernández, Jing Cui, Daniel E. Martínez-Tong, Aurora Nogales

**Affiliations:** 1 Instituto de Estructura de la Materia, IEM-CSIC, C/Serrano 121, 28006 Madrid, Spain; edgar.gutierrez@ehu.eus (E.G.-F.); jingcui@zzuli.edu.cn (J.C.); danielenrique_martineztong001@ehu.eus (D.E.M.-T.); 2 POLYMAT, University of the Basque Country (UPV/EHU), Av. de Tolosa 72, 20018 San Sebastian, Spain; 3 Key Laboratory of Surface and Interface Science of Henan Province, School of Material and Chemical Engineering, Zhengzhou University of Light Industry, 136, Science Avenue, Zhengzhou 450002, China; 4 Departamento de Polímeros y Materiales Avanzados: Física, Química y Tecnología, University of the Basque Country (UPV/EHU), 20018 San Sebastian, Spain

**Keywords:** water-based polymer inks, functional polymer, semicrystalline structure

## Abstract

In this study, water-based functional polymer inks are prepared using different solvent displacement methods, in particular, polymer functional inks based on semiconducting polymer poly(3-hexylthiophene) and the ferroelectric polymer poly(vinylidene fluoride) and its copolymers with trifluoroethylene. The nanoparticles that are included in the inks are prepared by miniemulsion, as well as flash and dialysis nanoprecipitation techniques and we discuss the properties of the inks obtained by each technique. Finally, an example of the functionality of a semiconducting/ferroelectric polymer coating prepared from water-based inks is presented.

## 1. Introduction

Functional polymers with applications in organic electronics have attracted continuous attention in recent years. These materials combine the beneficial properties of polymers (lightweight, flexibility, and low cost) with the functionality required for electronic applications. For example, applications of functional polymers have included semiconducting components and piezoelectric elements, among several others [1,2,3,4]. Improvements in solubility and processability of these polymers have boosted their evolution and relevance, in contrast to the original simpler conjugated polymers such as polyaniline and polythiophene [5]. In fact, this second generation of semiconducting polymers has allowed the fabrication of thin and flexible layers with semiconducting properties, by simply depositing a solution of the functional polymer on a flexible substrate [5]. These layers constitute the basis of electronic devices with an organic functional component, such as organic solar cells (OSCs) [6], organic light-emitting diodes (OLEDs) [7], and organic-field effect transistors (OFETs) [8].

Together with semiconducting polymers, ferroelectric polymers are considered to be key for the future of flexible organic electronics [9]. Ferroelectric polymers are functional materials that possess spontaneous polarization that is switchable with an electric field [10,11]. This functionality has encouraged research directed to the use of ferroelectric polymers as active materials in sensors [12], non-volatile memory [9,13], and nanogenerators for energy harvesting [14,15]. Ferroelectric polymers have also been studied in combination with semiconducting polymers for potential applications such as low-voltage organic diodes [16], non-volatile memory [17,18], and organic solar cells [1].

Although these materials are currently used for the development of eco-friendly devices, they are not water soluble, and thus require the use of halogenated and aromatic solvents. In turn, the fabrication of polymer devices from solvent-based protocols represents an important environmental issue. As an alternative, water-based functional polymer inks are a greener and environmentally friendly approach. These inks are generally formed by a dispersion of polymers in water [19,20], with the dispersed part inherently nanostructured. In most cases, inks are composed of water-dispersed polymer nanospheres with diameters ranging between 50 and 500 nm [21,22]. These polymeric nanoparticles and their dispersions have applications in many scientific fields including photonic systems [4], adhesives [23], as well as biomedical applications such as targeting [24], molecular imaging [25], and drug delivery activated by external stimuli [26,27] and organic electronics [28]. In addition to the advantage of using water as the primary continuous phase, functional inks composed of polymer nanoparticles offer a highly effective area and size and structural tunability by playing with preparation conditions [21,29,30]. The formation of nanoparticles for water-based inks can also be carried out using “preformed” polymers [19], i.e., commercially available systems with low polydispersity index, high purity, and traceability. Using these sorts of polymers as precursors allows the a prior knowledge of their bulk properties, and guarantees the absence of chemical residues from the polymerization processes, a common problem observed in the case of nanoparticle polymer synthesis [31,32].

**Figure 1:**
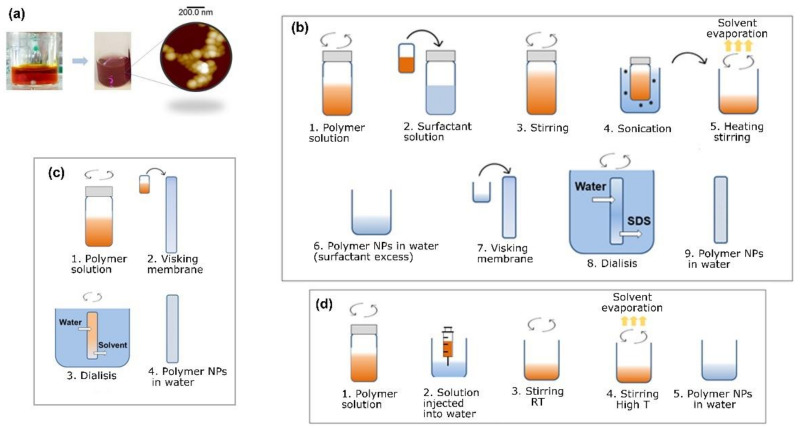
(**a**) Example of water-based ink preparation of poly(3-hexylthiophene-2,5-diyl) (P3HT). Starting from a P3HT solution in an organic solvent (red liquid), a water-based ink containing polymer nanoparticles is prepared (pink liquid). The detailed morphology of the polymer nanoparticles obtained by atomic force microscopy (AFM) is shown; (**b**–**d**) schematics illustrating the preparation steps for water-based inks of functional polymer nanoparticles by miniemulsion, dialysis nanoprecipitation, and flash nanoprecipitation, respectively.

In general, the fabrication of water-based inks from preformed bulk polymers involves a controlled precipitation of polymer solutions by exchanging a good organic solvent with a bad solvent or antisolvent [33]. Polymer chains in good solvents adopt an expanded random coil conformation [34], and the addition of a bad solvent decreases the solubility of the media which affects the polymer chain conformation. In this situation, the polymer adopts a globular envelope, which reduces the effective interfacial area with the liquid and leads to total separation of phases if the solute is fully insoluble. Depending on the interaction between the good and bad solvents, it is possible to freeze the globular conformation using different approaches. We explored three different techniques for preparing water-based inks, namely miniemulsion, dialysis nanoprecipitation, and flash nanoprecipitation. A schematic of each one of these preparation techniques is shown in Figure 1.

The general basis of the miniemulsion technique [19], also known as miniemulsion/solvent evaporation [35], consists of preparing a polymer solution and mixing it with water in the presence of a surfactant. The technique is schematically described in Figure 1b. The following conditions must be fulfilled: (1) the polymer solvent (good solvent) must be immiscible with water (bad solvent) and (2) the polymer should not dissolve in water, so that an emulsion can be formed after mixing. Miniemulsion involves applyig high-energy sonication to the emulsion using an ultrasonicator. The boiling point should be below 100 °C. By heating the emulsion formed by the polymer solution and the water dissolved surfactant, the solvent is evaporated while continuously stirring, leading to solid polymer particles stabilized by the surfactant in water. Previously, water-based inks of poly(ethyl methacrylate) (PEMA), poly(styrene) (PS), and poly(bisphenol A carbonate) have been prepared using this method [21,29], as well as semiconducting polymers such as methyl substituted lad-der-type poly(para-phenylene) (Me-LPPP), polyfluorene (PF) derivatives, and polycyclopentadithiophenes (PCPDTs) [19,36].

In contrast to miniemulsion, the flash and dialysis techniques do not use surfactants. These protocols rely on crashing out hydrophobic polymer chains by displacing a solvent with a non-solvent. To obtain water-based inks, the polymer solvent should be water-miscible organic molecules, while water acts as a poor solvent. Regarding the miniemulsion technique, surfactant-free techniques are regarded as post-polymerization dispersion methods, since bulk polymer chains of known molecular weight are used as precursors. However, surfactant-free techniques present certain advantages. For example, the nanoparticle diameter depends mostly on the polymer solution concentration and there are no sources of residual contaminants that should be removed (as excess of surfactant), therefore, the long step of surfactant removal is avoided.

Flash nanoprecipitation is a simple and practical technique to obtain water-based inks. In this case, the polymer solution and the non-solvent are rapidly mixed, for example, using a syringe, while stirring continuously with a water-miscible solvent. After mixing, the organic solvent is removed by heating the dispersion above the solvent’s boiling point, which should be lower than that of water. A schematic of the overall process is shown in Figure 1c.

The flash nanoprecipitation technique is not suitable when the water-miscible solvent has a higher boiling temperature than water. In these situations, it is possible to obtain nanoprecipitated polymer particles by slowly dialyzing the polymer solution against water [37] using the so-called dialysis nanoprecipitation technique (Figure 1d). In general, a polymer solution is prepared using a water-miscible solvent and poured into a dialysis membrane. Then, the filled membrane, previously rinsed with the organic solvent, is dialyzed against water, where, due to the miscibility of the liquids and the size threshold of the membrane walls, the solvent molecules exit the membrane into the water bath, and the water molecules are replaced until chemical equilibrium is reached. During the solvent displacement (i.e., bad solvent enters into the membrane), a gradual decrease in the solubility of the polymer produces the collapse of the polymer chain and, in this way, a homogeneous colloid of the polymer in water is formed. After several dialysis cycles, the total removal of the organic solvent is achieved.

In this study, we aim to show how different techniques can be applied for obtaining functional water-based inks, i.e., paradigmatic semiconducting polymer poly(3-hexylthiophene-2,5-diyl) (P3HT) ink, a blend of P3HT with fullerene-based material [6,6]-phenyl C_71_ butyric acid methyl ester (PC_71_BM) ink, and poly(vinylidene fluoride) (PVDF) and poly(vinylidene fluoride-co-trifluoroethylene) P(VDF-TrFE) ink. In each case, the preparation technique is chosen taking into account the desired properties of the resulting ink and the solubility and properties of the polymer solvent. P3HT inks were prepared using flash and miniemulsion techniques; PCBM ink was prepared using the miniemulsion technique; PVDF ink was prepared using the dialysis nanoprecipitation technique; and P(VDF-TrFE) ink was prepared using the flash precipitation technique.

We explored the size and crystallinity of these systems using conventional methods such as atomic force microscopy and X-ray scattering and its functional properties with cutting edge methods such as piezoresponse force microscopy. Our experimental results provide structural properties, while simultaneously measuring physical properties of interest for functionality. Finally, we present examples of applications of our water-based polymer inks.

## 2. Materials and Methods

### 2.1. Materials

Poly(3-hexylthiophene-2,5-diyl) (P3HT) was purchased from Ossila Ltd. (batch no. M102, Sheffield, UK). The polymer had an average molecular weight of 65 kg/mol, a polydispersity index of 2.2, and regioregularity of 95.7%, according to the manufacturer. [6,6]-Phenyl C71 butyric acid methyl ester (PC71BM) was purchased from Ossila Ltd. (batch no. M114, purity > 99%). Poly(vinylidene fluoride) (PVDF) was purchased from Sigma-Aldrich (batch no. 182702, St. Louis, MO, USA), with an average molecular weight of 534 kg/mol, according to the manufacturer. Poly(vinylidene fluoride-co-trifluoroethylene) P(VDF-TrFE) was purchased from Piézotech S.A.S. (Hésingue, France), with a VDF/TrFE mol ratio 24/76, according to the manufacturer. A PEDOT/PSS aqueous dispersion (Heraeus Clevios™ AI 4083, PEDOT/PSS ratio 1:6, Ossila, Sheffield, UK) was used to prepare thin films on doped silicon wafers. The substrates were sonicated in acetone for 10 min, and then in isopropyl alcohol for another 10 min before use and, subsequently, they were rinsed in deionized water. Finally, the substrates were dried with nitrogen blow.

For dialysis, we used a dialysis tubing membrane (Visking DTV, Medicell Int Ltd. London, UK). The cutoff range was 12,000–14,000 g/mol and the tube diameter = 25.5 mm.

Sodium dodecyl sulfate (SDS) was purchased from Sigma-Aldrich (ACS reagent grade, St. Louis, MO, USA).

For the different preparations, the following solvents were used: chloroform (CHCl_3_) (purity higher than 99.98%) (Quimipur SLU, Madrid, Spain); tetrahydrofuran (THF) (EMPARTA ACS, purity > 99.5%, Merck, Darmstadt, Germany); and dimethyl acetamide (DMA) (Sigma-Aldrich, St. Louis, MO, USA) (purity 99.8%). All polymers and reagents were used as received. For all preparations, distilled water was used.

Two different types of functional semiconducting films with ferroelectric particles embedded were prepared. In one case, the continuous phase was PEDOT/PSS. To obtain these films, it was necessary to prepare an ink with nanoparticles and continuous phase. In the case of PEDOT-PSS, a 2:1 mixture of water-dispersed PEDOT-PSS and water-dispersed P(VDF-TrFE) NPs was prepared. This ink was deposited either by spin coating or by inkjet printing.

Spin coating was performed using a spin processor (Laurell WS-650 Series, Laurell Technologies Corporation, North Wales, PA, USA). The inks were dropped with a syringe on the substrate with a constant rotational speed of 3000 rpm, for 60 s, and an acceleration of 3000 rpm·s^−1^.

### 2.2. Techniques

Atomic force microscopy (AFM) measurements were carried out using a Multimode 8 equipment, controlled with Nanoscope V electronics (Bruker, Kasruhe, Germany). Topography images were taken in tapping mode, using gold-coated silicon probes (Tap300GB-G probes, BudgetSensors, Sofia, Bulgaria) (f_res_ = 300 kHz, k = 40 N/m). The water-based inks were deposited onto SiO_x_ wafers by spin coating (3000 rpm for 2 min). The SiOx substrates were thoroughly washed using acetone, isopropanol and, subsequently, air dried. PFM measurements were carried out by means of the same AF equipment in the piezoresponse mode. The topography and the ferroelectric signals were acquired simultaneously. In this case, a microscope was used in the contact mode, with a low deflection set point (0.3 V) in order to avoid damaging the samples. Samples deposited on conductive silicon were attached to metallic sample holders by using either conductive epoxy (CW2400 Chemtronics, Kannesaw, GA, USA) or double-sided carbon conductive tape (Ted Pella, Redding, CA, USA). Conductive PtIr-covered probes (model SCM-PIT, Bruker, Kasruhe, Germany) (k = 3 N/m) for PFM measurements were used. Hysteresis cycles were recorded using PFM, applying a tip bias ramp from −10 to 10 V at a frequency of 0.1 Hz. Wide angle X-ray scattering was either in transmission geometry or in grazing incidence geometry by using synchrotron light at beamline NCD_SWEET (Synchrotron ALBA, Cerdanyola del Vallés, Spain). In transmission geometry, the dried NPs were located perpendicular to the synchrotron beam, and the scattering intensity was collected in a LX255-HS detector (Rayonix, Evanston, IL, USA) located at 12.5 cm from the sample. For the grazing-incidence experiments, the scattering patterns were collected using this same detector. The water-based ink was drop casted onto a silicon wafer, and allowed to dry for several days. The incidence angle for the X-rays was 0.2° and the sample to detector distance was 14 cm. In both cases the X-ray wavelength used was 0.1 nm.

### 2.3. Description of the Techniques Used for the Preparation of Water-Based Inks

#### 2.3.1. Water-Based Inks Prepared by Miniemulsion

**Figure 2:**
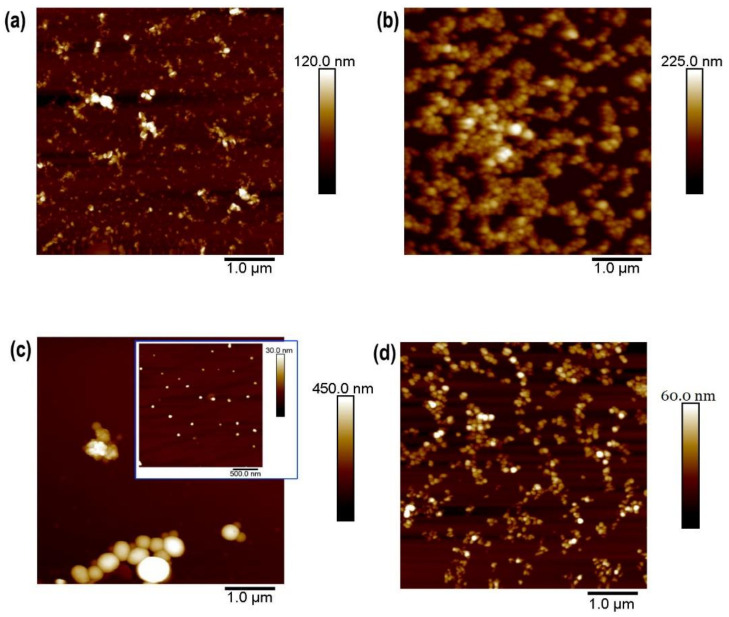
Examples of nanoparticles composing water-based inks, prepared by different techniques. AFM topography for (**a**) P3HT nanoparticles prepared by miniemulsion; (**b**) poly(vinylidene fluoride-co-trifluoroethylene) (P(VDF-TrFE)) nanoparticles prepared by flash nanoprecipitation; (**c**) poly(vinylidene fluoride) (PVDF) nanoparticles prepared by dialysis nanoprecipitation. The inset shows the amplificated z scale of the same image to highlight the presence of a bimodal size distribution of nanoparticles; (**d**) [6,6]-phenyl C_71_ butyric acid methyl ester (PC_71_BM) nanoparticles prepared by miniemulsion.

In this study, water-based inks were prepared from P3HT and PC_71_BM using the miniemulsion technique. Figure 1b shows a schematic of the overall process. For the preparation of P3HT nanoparticles, a 3 g/L P3HT solution in CHCl_3_ was prepared and heated at 40 °C for 1 h. Then, the polymer solution was mixed with a water solution of SDS at different concentrations. The emulsion formation was promoted by stirring vigorously for two hours at room temperature. Afterwards, miniemulsion was obtained by ultrasonication for 15 min, using an ultrasonic bath (power = 480 W) at room temperature. Then, to evaporate the CHCl_3_, the mixture was heated at 70 °C with continuous stirring, for one hour. Excess SDS was removed by dialyzing against distilled water, for 2 days, with several and continuous water exchanges. A water-based ink from P3HT, using miniemulsion, was prepared using P3HT nanoparticles, whose dried morphology was characterized by atomic force microscopy (AFM). Figure 2a shows AFM topography images of the P3HT nanoparticles deposited on silicon wafers. The surface topography was formed by aggregates of P3HT nanostructures, presenting a randomly closed packed (RCP) array, similar to the one previously reported by Holmes and collaborators for P3HT nanoparticulated films [38]. It is worth noting that the P3HT nanoparticles did not always present a spherical shape. Instead, some particles presented sharp edges and straight sides, closely resembling rectangles. This fact has been previously attributed to the presence of crystalline domains within the nanoparticles [38].

A water-based ink from PC_71_B nanoparticles was also successfully prepared using miniemulsion, which is discussed later. The preparation protocol was the same as that described for P3HT, but PC_71_BM was dissolved in chloroform (3 g/L), stirring the mixture at room temperature for 1 h. The water-based ink was formed by a dispersed PC_71_BM colloid.

#### 2.3.2. Water-Based Inks: Surfactant-Free Methods

##### Flash Nanoprecipitation

An example of functional polymer inks prepared using the flash nanoprecipitation technique is shown in Figure 2b. The specific protocol to prepare P(VDF-TrFE) water-based inks by flash nanoprecipitation was the following: A 2 g/L polymer solution in THF was prepared. The solution was left under stirring for 2 h, at 60 °C, to promote polymer solubility. After cooling to room temperature, the solution was injected into a water reservoir (ten times the volume of the solution), both liquids at the same temperature, and immediately started stirring at 900 rpm for 15 min. After the initial stirring step, a slow stirring rate was maintained and the mixture was heated to 70 °C to remove the THF (30–60 min). It was also possible to prepare P3HT inks using this technique. In this case, the starting point was a 3 g/L P3HT solution in THF. The rest of the protocol was the same as the one described above for P(VDF-TrFE) ink. The formation of the dispersion was clearly observed immediately after injecting the solution into water, by a sudden color change (see Figure 1a).

##### Dialysis Nanoprecipitation

Figure 2c shows an example of PVDF nanoparticles prepared by dialysis nanoprecipitation. For this particular case, a clear bimodal size distribution was obtained, as highlighted in the inset of Figure 2c.

PVDF water-based inks were prepared by dissolving the polymer in DMA at 2 g/L, at room temperature. The solution was dialyzed against distilled water using a dialysis membrane, previously cleaned and rinsed in DMA. The dialysis took around twenty-four hours, during which water was changed periodically. Figure 2c shows an example of the morphology of the obtained PVDF nanoparticles by this dialysis nanoprecipitation technique. Similarly, ferroelectric polymer water-based inks were prepared by dissolving P(VDF-TrFE) at 2 g/L in DMA at 40 °C, for 3 h. After cooling to room temperature, we followed a similar dialysis nanoprecipitation protocol as the one just described for PVDF.

Table 1 summarizes the protocols and the conditions for preparation of different functional polymer water-based inks.

## 3. Results and Discussion

### 3.1. Semiconducting Polymer Water-Based Inks

Miniemulsion has been one of the techniques of choice for preparing water-based inks of semiconducting polymers [19,39,40,41,42,43,44] and it implies the use of surfactant. Besides stabilizing the solid nanoparticles, the surfactant may impact the final properties of the ink. In this study, we used different techniques to prepare inks whose functional component was an organic semiconducting material. In addition, by choosing the appropriate conditions, it was possible to prepare water-based inks in which the solid dispersed particles were intrinsically a blend of a donor acceptor nanoparticles.

#### Inks from P3HT

**Figure 3:**
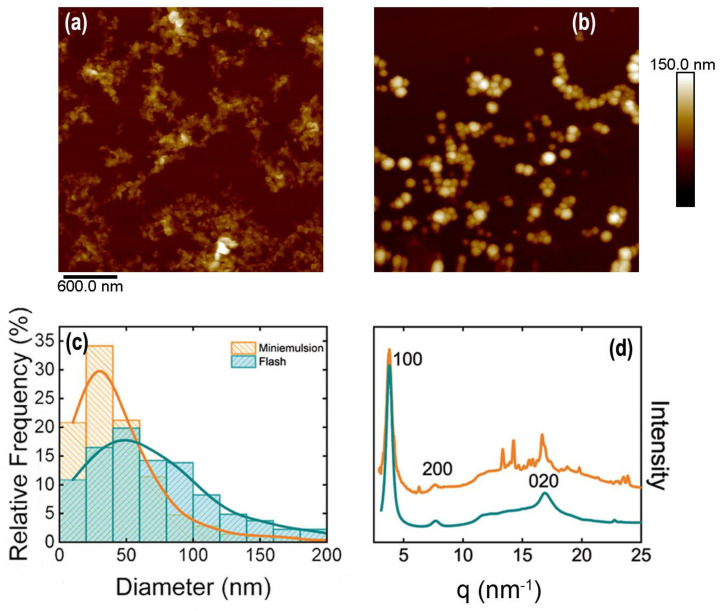
Morphology of spin-coated miniemulsion (**a**) and flash (**b**) P3HT inks onto silicon wafers, observed by AFM; (**c**) size distribution of the observed nanoparticles; (**d**) X-ray diffraction profile of both types of P3HT nanoparticles.

Miniemulsion and flash techniques are both suitable for the preparation of P3HT water-based inks. We have recently shown that the photophysical properties of the P3HT-based inks depended on the preparation technique. In the case of miniemulsion P3HT-based inks, the interactions between SDS and P3HT are critical for the control of P3HT semicrystalline structure [30]. Figure 3 shows the topography of P3HT nanoparticles prepared either by the miniemulsion technique (Figure 3a) or by the flash technique (Figure 3b). The inks were both prepared starting from P3HT solutions with the same polymer concentration. Despite this, nanoparticles from miniemulsion precipitation are smaller with a narrower size distribution than those from flash preparation (see size histogram in Figure 3c). Miniemulsion nanoparticles are not spherical, and they exhibit some sharp, straight borders, or even an almost rectangular shape and, when deposited by spin coating, tend to form aggregates. This feature has been associated with the presence of semicrystalline P3HT domains within the nanoparticles [38].

Nanoparticles prepared by flash (Figure 3b) are close to spheres. However, this does not necessarily imply that flash nanoparticles are not semicrystalline. In fact, the diffraction patterns obtained from both types of P3HT nanoparticles (Figure 3d) show the reflections corresponding to the (100) plane of the P3HT unit cell (q = 3.8 nm^−1^), higher orders of the same family of planes, and the (020/002) reflection (q ~ 17 nm^−1^). P3HT unit cell (h00) reflections are associated with the lamellar stacking (with spacing d = 1.65 nm). The (020) reflection is due to chain stacking along the molecular orbitals, and (002) reflection derived from the periodicity along the conjugated main chain [45]. In the case of miniemulsion nanoparticles, the diffraction pattern, in addition to the typical Bragg peaks from P3HT, additional sharp peaks appear in the region between q = 10 nm^−1^ and q = 15 nm^−1^ that, in a first approach, can be attributed to SDS. Recently, it has been shown that these Bragg peaks observed in miniemulsion P3HT nanoparticle diffraction patterns arose from a hybrid phase formed by the SDS surfactant and P3HT, which is the phase responsible for the different photophysical behaviors observed in both types of particles [30].

The type and amount of surfactant can also affect the shape, and hence the properties of the particles prepared by miniemulsion [46,47]. The concentration of the surfactant in water also significantly influences the nanoparticles size. As a general rule, a higher SDS concentration in water allows coating larger polymer-water interfaces leading to formation of smaller particles [48,49].

**Figure 4:**
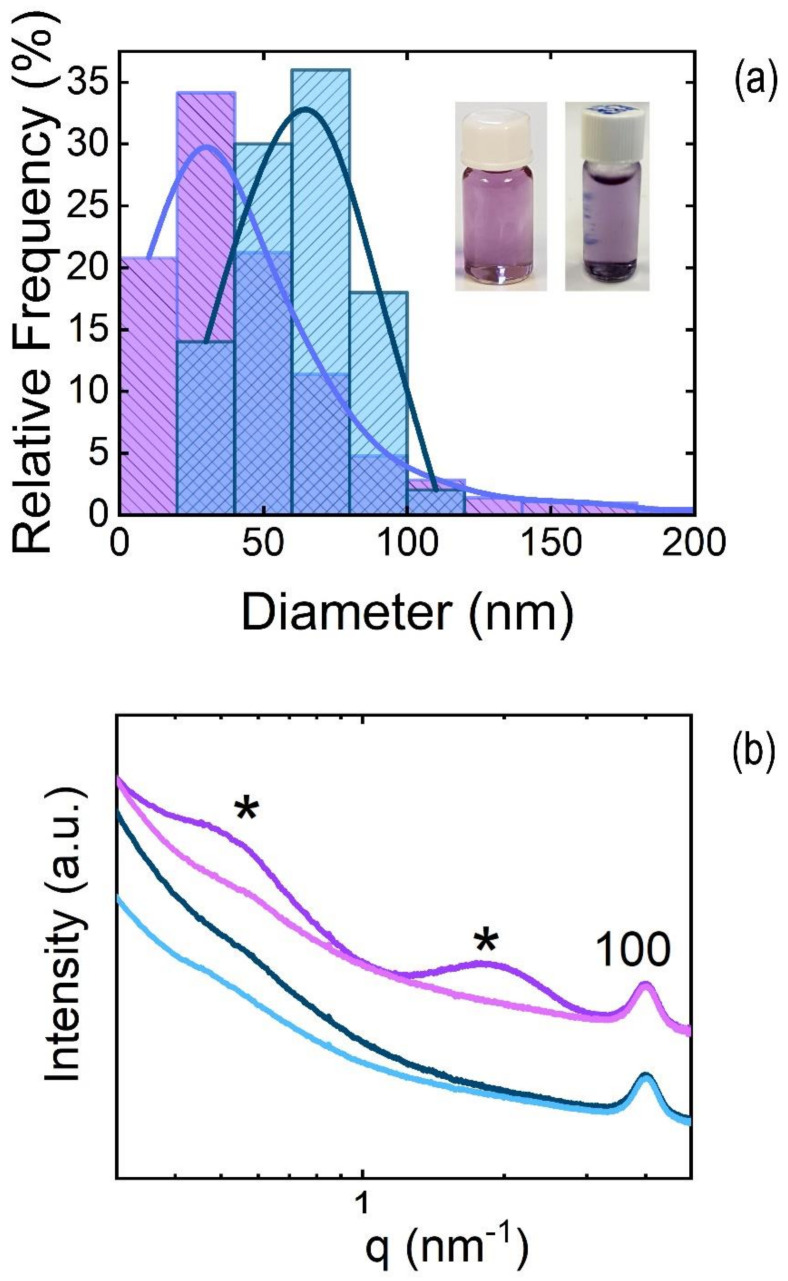
(**a**) Particle diameter histogram from P3HT particles prepared by miniemulsion using two different concentrations of sodium dodecyl sulfate (SDS), i.e., 0.1% in weight (blue bars) and 1% in weight (purple bars). Images of the inks prepared by the two concentrations are shown in the inset. Ink prepared using 1% wt. SDS concentration (left bottle) and 0.1% wt. SDS concentration (right bottle); (**b**) SAXS from the inks before (darker curves) and after dialysis (lighter curves). 1% wt. SDS purple curves and 0.1% wt. SDS blue curves. Asterisks mark the features corresponding to the SAXS curves of SDS micelles.

When using a concentration of SDS below the critical micellar concentration (CMC), the obtained P3HT particles are considerably larger (Figure 4a). Small angle X-ray scattering performed directly on the water-based miniemulsion inks, before and after dialyzing, reveals the presence of SDS micelles in the case of the 1% SDS ink. The SAXS feature characteristics of SDS micelles disappear after dialyzing. However, SAXS curves from the sample prepared with a 0.1% SDS water solution do not show the typical scattering from SDS micelles, but only an excess of scattering that is removed after dialysis (Figure 4b). In addition to this fact, the photophysical properties of the water-based P3HT inks prepared by this technique are similar, as can be evidenced by their characteristic blue color as compared with the red shifted color exhibited by the flash nanoparticle inks.

**Figure 5:**
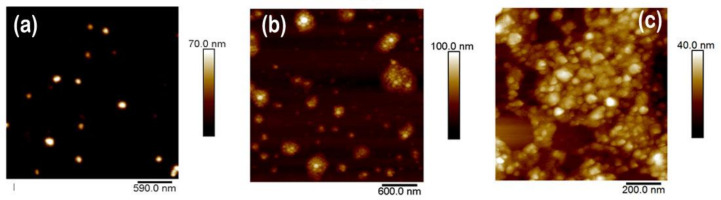
Morphology of particles deposited onto silicon wafers. (**a**) PC_71_BM nanoparticles prepared by flash technique; (**b**) PC_71_BM nanoparticles prepared by miniemulsion; (**c**) nanoparticles of a 1:1 blend of P3HT and PC_71_BM prepared by miniemulsion.

Using the miniemulsion and flash techniques, it is also possible to prepare aggregates of PC_71_BM molecules in the form of nanoparticles that, afterwards, can be used to prepare controlled nanophase separated blends with P3HT to form the paradigmatic heterojunction for the active layers (Figure 5a,b). On the one hand, using miniemulsion, it is also possible to prepare nanoparticles from a blend (Figure 5c), in which both components of the blend are found in each particle [50]. On the other hand, sequential flash nanoprecipitation allows the preparation of core-shell nanoparticles with a donor core and an acceptor shell [47]. In the present case, different techniques produce nanoparticles with different size distributions. Table 2 shows the characteristic parameters of the obtained size distribution of the nanoparticles of PC_71_BM and blends of PC_71_BM and P3HT prepared under different conditions.

Our results indicate that, for the same starting solution concentration, the flash nanoprecipitation technique produces larger aggregates than the miniemulsion technique. This trend was also observed for P3HT, but in the case of PC_71_BM the size difference between both techniques is larger than in the case of P3HT. In the case of nanoparticles formed by miniemulsion from a 1:1 P3HT/PC_71_BM, the size of the obtained nanoparticles is similar to that obtained for nanoparticles of P3HT.

### 3.2. Ferroelectric Polymer Water-Based Inks

#### 3.2.1. PVDF Nanoparticles

Recently, significant study efforts have been directed to include PVDF in organic electronics technologies, since PVDF can exhibit a large piezoelectric and ferroelectric response, due to the net dipole of polymer chains. However, PVDF can crystallize on different phases which are not all polar, and thus ferroelectric. Looking at miniaturization of ferroelectric devices, PVDF nanoparticles have been prepared by dialysis procedure, and their morphology, inner structure, and thermal behavior have been characterized.

**Figure 6:**
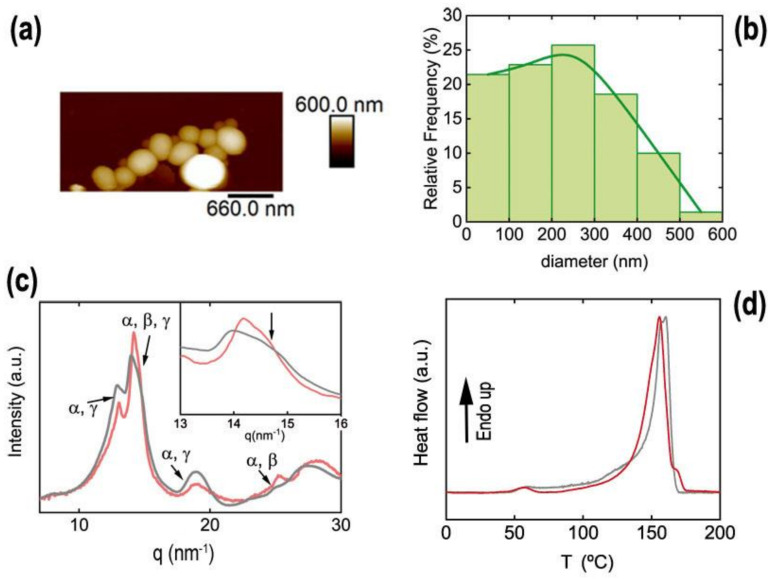
(**a**) Topographical image taken by AFM of PVDF nanoparticles prepared by dialysis technique; (**b**) histogram for the measured diameters; (**c**) X-ray diffracted intensity profiles for bulk PVDF processed from dimethyl acetamide (DMA) solution (black curve) and PVDF nanoparticles (red curve). Both diffractograms are normalized by setting the intensity maximum of this q range as 1 and the minimum as 0. The inset is a magnification of the zone of the most intense peak. The labels indicate the possible crystalline phases that would have a reflection in that particular position; (**d**) differential scanning calorimetry (DSC) heating ramps of PVDF film dried from DMA solution (black curve) and PVDF nanoparticles (red curve).

PVDF nanoparticles were prepared using the dialysis technique as described in Section 2, using the concentrations reflected in Table 1. The size and the height of PVDF nanoparticles were characterized by AFM on samples deposited by drop-casting on silicon wafers (Figure 6a). It can be observed that the morphology of the obtained nanoparticles is nearly spherical, with a mean diameter around 230 nm and standard deviation of 125 nm, as determined by measuring the size of 70 nanoparticles observed by AFM. A histogram of the PVDF nanoparticle diameters is shown in Figure 6b.

X-ray diffraction provides evidence that the nanoparticles are semicrystalline. In order to compare the crystalline structure with that of a bulk PVDF sample, a film was prepared by drying a PVDF solution in DMA by drop casting. The diffractograms of both the film and the nanoparticles (Figure 6c, black and red curve, respectively) share similar features with intense peaks between q = 12 nm^−1^ and q = 15 nm^−1^, a broad peak at q ≈ 19 nm^−1^, and a broad peak around q = 28 nm^−1^. However, there are also some subtle differences such as a weak peak at q ≈ 25 nm^−1^ only appreciated in the diffractogram from the nanoparticles (Figure 6c, red curve). Labels were added to indicate the possible crystalline origin of the peaks that will be unraveled [51,52,53,54]. The main reflections observed in the diffractograms from the film sample and the nanoparticles correspond predominantly to α, γ, or both phases [55]. Samples with almost pure content in α phase present two peaks between q = 12 nm^−1^ and q = 13 nm^−1^ associated with (100) and (020) reflections, respectively [52]. The (020) reflection of γ phase is also located in this region (q = 13.1 nm^−1^) [52]. The maximum of intensity in both diffractograms occurs around q ≈ 14.1 nm^−1^, which is near the α (110) reflection (q = 14.17 nm^−1^) [51]. However, as shown in the inset of Figure 6c, additionally this peak may have contributions from several reflections at higher q, such as the (110/101) reflections of γ phase [51] at q = 14.39 nm^−1^ or the (110/200) reflection from β phase [51] at q = 14.6 nm^−1^. The broad peak at q ≈ 19 nm^−1^ is commonly found in PVDF samples with a high α content, as it is associated with (021) reflection of this phase [51], however, the (022) reflection from γ phase is located at q ≈ 18.9 nm^−1^ [53]. The weak peak at q ≈ 25 nm^−1^, observed only in the nanoparticles diffractogram, is identified either as the (200) reflection of α phase [51] or (020) reflection from β phase [51,54]. The most common crystalline phases found in PVDF are apolar α phase and polar β and γ phases [52]. The shape of the most intense peak indicates that it is composed of more than one reflection (see inset of Figure 6c) from different phases. In fact, the width of the most intense peak around q = 14.6 nm^−1^ (marked with an arrow in the inset of Figure 6c) could indicate the presence of the (110/200) reflection from β phase [51] at q = 14.6 nm^−1^ in both diffractograms. The DSC experiments were performed on PVDF film casted from solution and dried at 100 °C and on PVDF nanoparticles (Figure 6d). Heating ramps of both PVDF systems show an endothermic peak around 160 °C, which is associated with the melting point of crystalline phases [56]. However, again, subtle differences in DSC traces from bulk PVDF and PVDF nanoparticles can be observed. The melting peak present in both samples around 160 °C can be attributed to the melting point of α and β phases [52], which in DSC are not distinguishable. In addition, a shoulder at 170 °C is observed in the DSC heating trace from the PVDF nanoparticles, which is not present in the case of the PVDF film. It has been reported that the γ phase presents a higher melting temperature than the α and β phases [52]. The DSC results indicate the presence of the polar γ phase within PVDF nanoparticles. The presence of polar phases (γ or β) in the sample of nanoparticles can be explained in terms of the interaction between the PVDF chains and molecules of the polar solvent used. PVDF was initially dissolved in DMA, which is a high polar solvent. The interaction between the NH_2_ group in DMA molecules and the fluorine atoms in PVDF through hydrogen bonds has been reported to be a precursor of the polar phases (β and γ) of the polymer [57,58]. This could be the origin of the polar phase in nanoparticles, since PVDF is crystallized from a solution in polar solvent, since the water molecules displaced the DMA molecules during the dialysis procedure.

#### 3.2.2. P(VDF-TrFE) Nanoparticles

P(VDF-TrFE) presents the spontaneous ferroelectric phase when it is deposited from solution or processed from the melt or from the solution [59]. Therefore, it offers the possibility of fabricating thin films and other nanostructures with ferroelectric behavior, and it has potential interest as a functional material in several dielectric and ferroelectric devices such as non-volatile memories [13,60] and piezoelectric sensors [61]. We prepared water-based inks containing P(VDF-TrFE) nanoparticles using the flash technique. The P(VDF-TrFE) solutions employed to fabricate nanoparticles were prepared following the conditions shown in Table 1.

**Figure 7:**
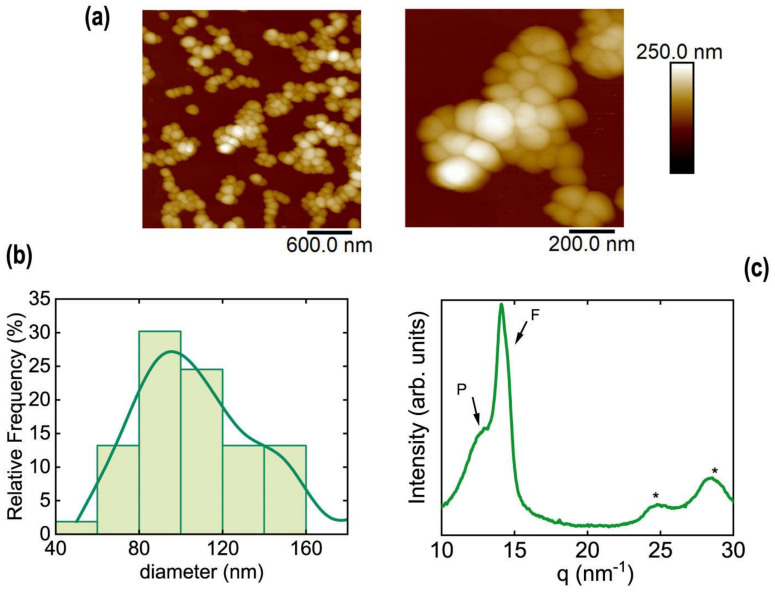
(**a**) Examples of AFM topography images of P(VDF-TrFE) nanoparticles prepared by flash nanoprecipitation; (**b**) histogram for the measured diameters of P(VDF-TrFE) nanoparticles prepared by flash; (**c**) diffractogram obtained from P(VDF-TrFE) nanoparticles prepared by flash and deposited by drop-casting. Most intense peaks associated with paraelectric (P) and ferroelectric (F) phase are labelled. Peaks marked with an * are associated to the ferroelectric phase.

The size distribution of nanoparticles measured by AFM (Figure 7a) is centered around 100 nm. The histogram for the measured diameter of P(VDF-TrFE) nanoparticles is shown in Figure 7b.

P(VDF-TrFE) nanoparticles are not perfectly spherical but show some irregular shapes. This could be associated with the semicrystalline nature of nanoparticles confirmed by X-ray diffraction (Figure 7c). The semicrystalline nature of nanoparticles is revealed by the following four features associated with phases of P(VDF-TrFE) (Figure 7c): a broad peak centered at q = 13.4 nm^−1^, an intense Bragg peak at q = 14.11 nm^−1^, and two peaks at q = 24.7 nm^−1^ and at q = 28.4 nm^−1^, marked with “*”. The broad peak at q = 13.4 nm^−1^ matches with the superposition of (110/200) reflections from the paraelectric crystalline phase of P(VDF-TrFE), whereas the intense narrow peak at q = 14.11 nm^−1^ contains the (110) and (200) reflections from the ferroelectric β phase [62]. This peak presents a bimodal shape (see inset in Figure 7c), with the most intense peak at q = 14.11 nm^−1^ and a shoulder at q = 14.5 nm^−1^ (downward arrow in the inset of Figure 7c). In addition, peaks marked with * are also reported to be reflections, due to the crystalline ferroelectric phase [62]. These results are in line with diffractograms from other P(VDF-TrFE) nanostructures [22,63].

##### Ferroelectric Ink Functionality

**Figure 8:**
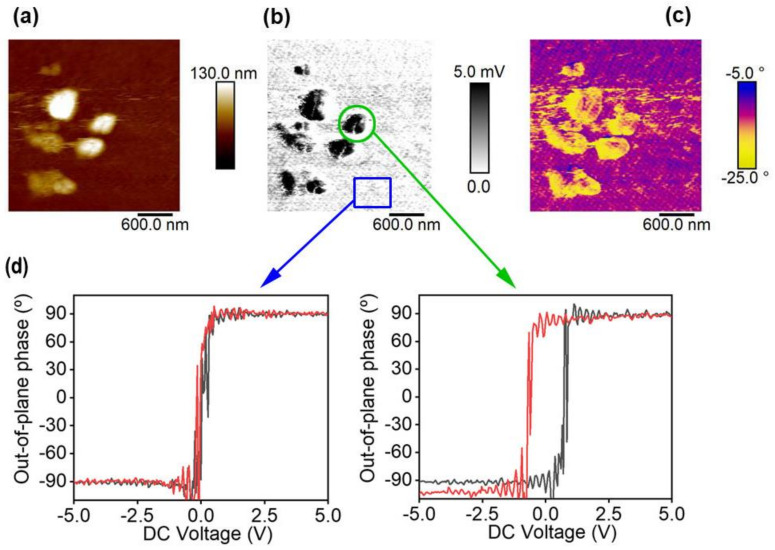
(**a**) Topography image in contact mode of PEDOT/PSS/(P(VDF-TrFE) nanoparticles; (**b**) piezoelectric amplitude; (**c**) piezoelectric phase in the out-of-plane direction; (**d**) piezoelectric phase in the out-of-plane direction measured applying a DC bias = −5 V → 5 V → −5 V over a flat zone associated with PEDOT/PSS film (blue square in (**b**) and on one of the protuberances associated with PVDF-TrFE nanoparticles embedded in the film (green circle in (**b**)).

As an example of the functionality of the water-based inks, we fabricated a polymer-polymer composite using the PVDF-TrFE nanoparticles presented in the previous section. The polymer nanoparticles were embedded within a semiconducting polymer matrix, in this case PEDOT/PSS, which could be obtained in the form of an aqueous colloid. The polymer-polymer composite was prepared by mixing the PEDOT/PSS dispersion with the P(VDF-TrFE) nanoparticle aqueous dispersion with a volume relation of 1:1. The mixture was spin-coated onto a silicon wafer. AFM images of the sample of PEDOT/PSS/P(VDF-TrFE) nanoparticles are shown in Figure 8.

Figure 8a shows the topography image taken in contact mode. Figure 8b represents the piezoelectric amplitude in the out-of-plane direction, whereas Figure 8c represents the piezoelectric phase in the out-of-plane direction. In the topography image (Figure 8a) a continuous phase with some protuberances is observed. Compared with what is observed in the PFM amplitude image (Figure 8b), the continuous phase does not show any piezoelectric amplitude, whereas the protuberances exhibit piezoelectric amplitude in the vertical direction demonstrating its ferroelectric nature. Therefore, the protuberances are likely to be aggregates of nanoparticles embedded in the continuous phase, which is formed by PEDOT/PSS without P(VDF-TrFE) remains. Changes in colors on the phase image (Figure 8c) suggest that the nanoparticles present random orientations along the vertical direction. Loops of DC voltage were applied on the PEDOT/PSS domain and on the embedded nanoparticles (Figure 8d). It can be observed that the PEDOT/PSS conducting region does not show ferroelectric behavior, whereas the embedded P(VDF-TrFE) nanoparticles can be measured and show a characteristic ferroelectric loop.

## 4. Conclusions

In this study, water-based inks formed by dispersions of nanoparticles of different functional polymers were fabricated using several techniques, based on exchanging the solvent from the polymer solution in a good organic solvent to an aqueous colloid system formed by globules of ~100 nm diameter. The ink preparation technique is selected on the basis of the properties of the organic solvent used for each type of polymer, with the most important being the miscibility of the solvent with water. In the case of P3HT, more than one technique can be used. P3HT inks can be prepared either by miniemulsion or by flash nanoprecipitation techniques. The coatings produced by the inks were analyzed by AFM and by different characterization methods based on each respective functionality, for example, ferroelectricity as determined by PFM. In the case of P3HT inks, we observed that the miniemulsion technique produced smaller nanoparticles with the conditions used. The X-ray and calorimetry experiments revealed synergy between P3HT and the surfactant used in miniemulsion (SDS), which indicated the existence of a blended phase of P3HT with SDS molecules probably located at the surface of nanoparticles. Water-based PVDF ink was prepared using the dialysis nanoprecipitation technique. The X-ray diffraction experiments revealed the presence of polar phases β and γ within PVDF nanoparticles. The polar phases within the PVDF nanoparticles can be induced by the interaction between the polar solvent (DMA) and the fluorine atoms of PVDF. We also prepared inks with P(VDF-TrFE) nanoparticles using a flash nanoprecipitation technique. These nanoparticles presented higher content in the ferroelectric phase, as demonstrated by X-ray diffraction. A multifunctional ink was prepared by combining semiconducting inks with ferroelectric inks. This ink was deposited onto a silicon substrate. The coating presented piezoelectricity as revealed by PFM experiments in contact mode.

## Figures and Tables

**Table 1:** Materials and Preparation Methods of Different Functional Polymer Inks.

Material	Preparation Method	Solvent	Concentration
P3HT	Miniemulsion	ClCH_3_	3 g/L
P3HT	Flash	THF	3 g/L
PC_71_BM	Miniemulsion	ClCH_3_	3 g/L
PC_71_BM	Flash	THF	3 g/L
P3HT/PC_71_BM blend	Miniemulsion	ClCH_3_	3 g/L
P(VDF-TrFE)	Flash	THF	2 g/L
PVDF	Dialysis	DMA	2 g/L

**Table 2:** Characteristic parameters of the size distribution of PC_71_BM and PC_71_BM/P3HT blend nanoparticles prepared under different conditions.

	Solvent	Average Size
PC_71_BM flash	THF (3 g/L)	120 nm
PC_71_BM miniemulsion	ClCH_3_ (3 g/L)	61 nm
P3HT/PC_71_BM 1:1 blend miniemulsion	ClCH_3_ (3 g/L)	45 nm

## Data Availability

The data presented in this study are available on request from the corresponding author.

## References

[1] Asadi K., de Bruyn P., Blom P.W.M., de Leeuw D.M. (2011). Origin of the efficiency enhancement in ferroelectric functionalized organic solar cells. Appl. Phys. Lett..

[2] Kleemann H., Krechan K., Fischer A., Leo K. (2020). A Review of Vertical Organic Transistors. Adv. Funct. Mater..

[3] Wu J., Meng Y., Guo X., Zhu L., Liu F., Zhang M. (2019). All-polymer solar cells based on a novel narrow-bandgap polymer acceptor with power conversion efficiency over 10%. J. Mater. Chem. A.

[4] Fudouzi H., Xia Y. (2003). Photonic Papers and Inks: Color Writing with Colorless Materials. Adv. Mater..

[5] Reynolds J.R. (2014). Pi-conjugated polymers: The importance of polymer synthesis. Conjugated Polymers: A Practical Guide to Synthesis.

[6] Berger P.R., Kim M. (2018). Polymer solar cells: P3HT:PCBM and beyond. J. Renew. Sustain. Energy.

[7] Bhatnagar P.K. (2018). Organic Light-Emitting Diodes—A Review.

[8] Forrest S.R. (2004). The path to ubiquitous and low-cost organic electronic appliances on plastic. Nature.

[9] Chen X., Han X., Shen Q.-D. (2017). PVDF-Based Ferroelectric Polymers in Modern Flexible Electronics. Adv. Electron. Mater..

[10] Zapsas G., Patil Y., Gnanou Y., Ameduri B., Hadjichristidis N. (2020). Poly(vinylidene fluoride)-based complex macromolecular architectures: From synthesis to properties and applications. Prog. Polym. Sci..

[11] Li H., Wang R., Han S., Zhou Y. (2020). Ferroelectric polymers for non-volatile memory devices: A review. Polym. Int..

[12] Bae J.-H., Chang S.-H. (2019). PVDF-based ferroelectric polymers and dielectric elastomers for sensor and actuator applications: A review. Funct. Compos. Struct..

[13] Hu Z., Tian M., Nysten B., Jonas A.M. (2009). Regular arrays of highly ordered ferroelectric polymer nanostructures for non-volatile low-voltage memories. Nat. Mater..

[14] Kim H.S., Kim D.Y., Kim J., Kim J.H., Kong D.S., Murillo G., Lee G., Park J.Y., Jung J.H. (2019). Ferroelectric-Polymer-Enabled Contactless Electric Power Generation in Triboelectric Nanogenerators. Adv. Funct. Mater..

[15] Whiter R.A., Boughey C., Smith M., Kar-Narayan S. (2018). Mechanical Energy Harvesting Performance of Ferroelectric Polymer Nanowires Grown via Template-Wetting. Energy Technol..

[16] Li M., Stingelin N., Michels J.J., Spijkman M.-J., Asadi K., Beerends R., Biscarini F., Blom P.W.M., de Leeuw D.M. (2012). Processing and Low Voltage Switching of Organic Ferroelectric Phase-Separated Bistable Diodes. Adv. Funct. Mater..

[17] Van Breemen A., Zaba T., Khikhlovskyi V., Michels J., Janssen R., Kemerink M., Gelinck G. (2015). Surface Directed Phase Separation of Semiconductor Ferroelectric Polymer Blends and their Use in Non-Volatile Memories. Adv. Funct. Mater..

[18] Gutiérrez-Fernández E., Rebollar E., Cui J., Ezquerra T.A., Nogales A. (2019). Morphology and Ferroelectric Properties of Semiconducting/Ferroelectric Polymer Bilayers. Macromolecules.

[19] Landfester K., Montenegro R., Scherf U., Güntner R., Asawapirom U., Patil S., Neher D., Kietzke T. (2002). Semiconducting polymer nanospheres in aqueous dispersion prepared by a miniemulsion process. Adv. Mater..

[20] Feng L., Zhu C., Yuan H., Liu L., Lv F., Wang S. (2013). Conjugated polymer nanoparticles: Preparation, properties, functionalization and biological applications. Chem. Soc. Rev..

[21] Martínez-Tong D.E., Cui J., Soccio M., García C., Ezquerra T.A., Nogales A. (2014). Does the glass transition of polymers change upon 3D confinement?. Macromol. Chem. Phys..

[22] Martínez-Tong D.E., Soccio M., Sanz A., García C., Ezquerra T.A., Nogales A. (2015). Ferroelectricity and molecular dynamics of poly(vinylidenefluoride-trifluoroethylene) nanoparticles. Polymer.

[23] Rose S., Prevoteau A., Elzière P., Hourdet D., Marcellan A., Leibler L. (2014). Nanoparticle solutions as adhesives for gels and biological tissues. Nature.

[24] Kamaly N., Xiao Z., Valencia P.M., Radovic-Moreno A.F., Farokhzad O.C. (2012). Targeted polymeric therapeutic nanoparticles: Design, development and clinical translation. Chem. Soc. Rev..

[25] Xie C., Heumüller T., Gruber W., Tang X., Classen A., Schuldes I., Bidwell M., Späth A., Fink R.H., Unruh T. (2018). Overcoming efficiency and stability limits in water-processing nanoparticular organic photovoltaics by minimizing microstructure defects. Nat. Commun..

[26] Crucho C.I.C.C. (2015). Stimuli-Responsive Polymeric Nanoparticles for Nanomedicine. ChemMedChem.

[27] Tran N.T.D.D., Truong N.P., Gu W., Jia Z., Cooper M.A., Monteiro M.J. (2013). Timed-Release Polymer Nanoparticles. Biomacromolecules.

[28] Xiao Z., Dong Q., Sharma P., Yuan Y., Mao B., Tian W., Gruverman A., Huang J. (2013). Synthesis and Application of Ferroelectric P(VDF-TrFE) Nanoparticles in Organic Photovoltaic Devices for High Efficiency. Adv. Energy Mater..

[29] Martínez-Tong D.E., Soccio M., Sanz A., García C., Ezquerra T.A., Nogales A. (2013). Chain Arrangement and Glass Transition Temperature Variations in Polymer Nanoparticles under 3D-Confinement. Macromolecules.

[30] Gutiérrez-Fernández E., Ezquerra T.A., Rebollar E., Cui J., Marina S., Martín J., Nogales A. (2021). Photophysical and structural modulation of poly(3-hexylthiophene) nanoparticles via surfactant-polymer interaction. Polymer.

[31] Mastrorilli P., Nobile C.F., Suranna G.P., Corradi A., Leonelli C., Veronesi P. (2003). Morphological characterization of poly(phenylacetylene) nanospheres prepared by homogeneous and heterogeneous catalysis. Appl. Organomet. Chem..

[32] Asua J.M. (2002). Miniemulsion polymerization. Prog. Polym. Sci..

[33] Rao J.P., Geckeler K.E. (2011). Polymer nanoparticles: Preparation techniques and size-control parameters. Prog. Polym. Sci..

[34] Flory P., Volkenstein M. (1969). Statistical Mechanics of Chain Molecules.

[35] Staff R.H., Schaeffel D., Turshatov A., Donadio D., Butt H.-J., Landfester K., Koynov K., Crespy D. (2013). Particle Formation in the Emulsion-Solvent Evaporation Process. Small.

[36] Bag M., Gehan T.S., Algaier D.D., Liu F., Nagarjuna G., Lahti P.M., Russell T.P., Venkataraman D. (2013). Efficient charge transport in assemblies of surfactant-stabilized semiconducting nanoparticles. Adv. Mater..

[37] Zhang C., Chung J.W., Priestley R.D. (2012). Dialysis Nanoprecipitation of Polystyrene Nanoparticles. Macromol. Rapid Commun..

[38] Holmes N.P., Marks M., Cave J.M., Feron K., Barr M.G., Fahy A., Sharma A., Pan X., Kilcoyne D.A.L., Zhou X. (2018). Engineering Two-Phase and Three-Phase Microstructures from Water-Based Dispersions of Nanoparticles for Eco-Friendly Polymer Solar Cell Applications. Chem. Mater..

[39] Kietzke T. (2007). Recent Advances in Organic Solar Cells. Adv. Optoelectron..

[40] Holmes N.P., Burke K.B., Sista P., Barr M., Magurudeniya H.D., Stefan M.C., Kilcoyne A.L.D., Zhou X., Dastoor P.C., Belcher W.J. (2013). Nano-domain behaviour in P3HT:PCBM nanoparticles, relating material properties to morphological changes. Sol. Energy Mater. Sol. Cells.

[41] Ulum S., Holmes N., Darwis D., Burke K., David Kilcoyne A.L., Zhou X., Belcher W., Dastoor P. (2013). Determining the structural motif of P3HT:PCBM nanoparticulate organic photovoltaic devices. Sol. Energy Mater. Sol. Cells.

[42] Ameri M., Al-Mudhaffer M.F., Almyahi F., Fardell G.C., Marks M., Al-Ahmad A., Fahy A., Andersen T., Elkington D.C., Feron K. (2019). Role of Stabilizing Surfactants on Capacitance, Charge, and Ion Transport in Organic Nanoparticle-Based Electronic Devices. ACS Appl. Mater. Interfaces.

[43] Xie C., Tang X., Berlinghof M., Langner S., Chen S., Späth A., Li N., Fink R.H., Unruh T., Brabec C.J. (2018). Robot-Based High-Throughput Engineering of Alcoholic Polymer: Fullerene Nanoparticle Inks for an Eco-Friendly Processing of Organic Solar Cells. ACS Appl. Mater. Interfaces.

[44] Algaier D.D. (2015). Impact of Fabrication Parameters on the Internal Structure of Poly(3-hexylthiophene) Nanoparticles. Ph.D. Thesis.

[45] Brinkmann M., Wittmann J.C. (2006). Orientation of Regioregular Poly(3-hexylthiophene) by Directional Solidification: A Simple Method to Reveal the Semicrystalline Structure of a Conjugated Polymer. Adv. Mater..

[46] Tan B., Li Y., Palacios M.F., Therrien J., Sobkowicz M.J. (2016). Effect of surfactant conjugation on structure and properties of poly(3-hexylthiophene) colloids and field effect transistors. Colloids Surf. A Physicochem. Eng. Asp..

[47] Holmes A., Deniau E., Lartigau-Dagron C., Bousquet A., Chambon S., Holmes N.P. (2021). Review of Waterborne Organic Semiconductor Colloids for Photovoltaics. ACS Nano.

[48] Visaveliya N., Köhler J.M. (2014). Control of Shape and Size of Polymer Nanoparticles Aggregates in a Single-Step Microcontinuous Flow Process: A Case of Flower and Spherical Shapes. Langmuir.

[49] Satapathi S., Gill H.S., Li L., Samuelson L., Kumar J., Mosurkal R. (2014). Synthesis of nanoparticles of P3HT and PCBM for optimizing morphology in polymeric solar cells. Proceedings of the Applied Surface Science.

[50] Richards J.J., Whittle C.L., Shao G., Pozzo L.D. (2014). Correlating structure and photocurrent for composite semiconducting nanoparticles with contrast variation small-Angle neutron scattering and photoconductive atomic force microscopy. ACS Nano.

[51] Cai X., Lei T., Sun D., Lin L. (2017). A critical analysis of the α, β and γ phases in poly (vinylidene fluoride) using FTIR. RSC Adv..

[52] Martins P., Lopes A.C., Lanceros-Mendez S. (2014). Electroactive phases of poly(vinylidene fluoride): Determination, processing and applications. Prog. Polym. Sci..

[53] Esterly D.M., Love B.J. (2003). Phase transformation to beta-poly(vinylidenefluoride) by milling. J. Polym. Sci. Part B.

[54] Lei T., Cai X., Wang X., Yu L., Hu X., Zheng G., Lv W., Wang L., Wu D., Sun D. (2013). Spectroscopic evidence for a high fraction of ferroelectric phase induced in electrospun polyvinylidene fluoride fibers. RSC Adv..

[55] Gregorio R. (2006). Determination of the α, β, and γ crystalline phases of poly(vinylidene fluoride) films prepared at different conditions. J. Appl. Polym. Sci..

[56] Merlini C., Barra G.M.O., Medeiros Araujo T., Pegoretti A. (2014). Electrically pressure sensitive poly(vinylidene fluoride)/polypyrrole electrospun mats. RSC Adv..

[57] Salimi A., Yousefi A.A. (2004). Conformational changes and phase transformation mechanisms in PVDF solution-cast films. J. Polym. Sci. Part B Polym. Phys..

[58] Horibe H., Sasaki Y., Oshiro H., Hosokawa Y., Kono A., Takahashi S., Nishiyama T. (2014). Quantification of the solvent evaporation rate during the production of three PVDF crystalline structure types by solvent casting. Polym. J..

[59] Calleja F.J.B., Arche A.G., Ezquerra T.A., Santa Cruz C., Batallan F., Frick B., Cabarcos E.L. (1993). Structure and properties of ferroelectric copolymers of poly (vinylidene fluoride). Structure in Polymers with Special Properties.

[60] Kang S.J., Bae I., Shin Y.J., Park Y.J., Huh J., Park S.-M., Kim H.-C., Park C. (2011). Nonvolatile Polymer Memory with Nanoconfinement of Ferroelectric Crystals. Nano Lett..

[61] Hong C.C., Huang S.Y., Shieh J., Chen S.H. (2012). Enhanced piezoelectricity of nanoimprinted sub-20 nm poly(vinylidene fluoride-trifluoroethylene) copolymer nanograss. Macromolecules.

[62] Bellet-Amalric E., Legrand J.F. (1998). Crystalline structures and phase transition of the ferroelectric P(VDF-TrFE) copolymers, a neutron diffraction study. Eur. Phys. J. B.

[63] García-Gutiérrez M.-C., Linares A., Martín-Fabiani I., Hernández J.J., Soccio M., Rueda D.R., Ezquerra T.A., Reynolds M. (2013). Understanding crystallization features of P(VDF-TrFE) copolymers under confinement to optimize ferroelectricity in nanostructures. Nanoscale.

